# Research on influencing factors and correlation pathways of rural teachers’ retention in China

**DOI:** 10.3389/fpsyg.2025.1728628

**Published:** 2026-01-12

**Authors:** Fang Chen, Ye-Jun Li, Tie-Fang Liu

**Affiliations:** 1 Faculty of Educational Sciences, Hunan Normal University, Changsha, China; 2 Office of Academic Affairs, Hengyang Normal University, Hengyang, China; 3 Department of Physical Education and Research, Hunan Institute of Technology, Hengyang, China

**Keywords:** Chinese rural education, rural education, rural teachers, teacher attrition, teacher retention

## Abstract

**Introduction:**

Teacher retention in rural China remains a critical issue, with significant implications for educational equity and student achievement. Addressing the challenge of retaining qualified teachers in remote and economically underdeveloped regions is essential for ensuring high-quality education for rural students. This study investigates the factors influencing rural teacher retention and proposes a hierarchical model to elucidate their interrelationships.

**Methods:**

Employing grounded theory and interpretive structural modeling (ISM), this study conducted semi-structured interviews with 33 rural teachers who had taught in rural areas for more than three years. The grounded theory approach was used to construct a theoretical model of factors influencing teacher retention, while ISM was applied to analyze the hierarchical relationships and interconnection pathways among these factors.

**Results:**

This study identifies multiple interrelated factors influencing the retention of rural teachers, constructs a hierarchical model, and reveals that teacher retention is jointly affected by foundational, transitional, and direct factors. The model highlights the complex interactive relationship between material conditions and psychological needs in teachers’ decisions to remain in rural education.

**Discussion:**

The findings suggest that improving rural teacher retention requires a multifaceted approach that addresses both material and psychological needs. Social status, professional identity, and a sense of educational mission emerge as core driving forces behind teacher retention. The study challenges the notion that salary increases alone can universally resolve retention issues, emphasizing the importance of enhancing teachers’ social recognition, providing clear career development pathways, and fostering a supportive school environment. Differentiated retention strategies should be developed based on teachers’ gender, subject-specific characteristics, and family circumstances to better meet their distinct needs. These insights provide policymakers with a comprehensive theoretical foundation for designing more precise and effective intervention strategies to enhance rural teacher retention.

## 1 Introduction

Teacher retention has long been a critical issue in rural education. From the perspective of natural population movement, the mobility of teachers is a normal phenomenon accompanying the socio-economic development. However, in economically underdeveloped or remote and backward regions, the outflow rate of teachers significantly exceeds the inflow rate. This phenomenon has become a focal point of concern for many countries worldwide (Nguyen and Springer, 2021), including the United States (Sutcher et al., 2019; Hudson and Hudson, 2019), the United Kingdom (United Kingdom Department for Education, 2019), Sweden (See and Gorard, 2020), Australia (Department of Education, 2021), China (Jiang and Yip, 2024), Japan (Ono, 2024), and others. Consistent research findings underscore the pivotal role of teachers as the most significant school-related factor influencing student achievement (Smith-Sherrod, 2021). For rural students in remote areas, retaining teachers equates to preserving hope. In economically, educationally, and resource-wise disadvantaged rural regions, teachers with higher academic qualifications and broader perspectives represent the sole resource through which rural children can transcend their mountainous confines and venture into the wider world. Therefore, each country is actively seeking ways to retain rural teachers by integrating its specific circumstances.

Existing research has demonstrated that factors such as salary compensation, commuting convenience, regional development level, social welfare, and student quality are significant determinants influencing the retention of rural teachers (Echazarra and Radinger, 2019). Specifically, schools with high-performing principals, regular and supportive communication, and better facilities and resources are more conducive to retaining rural teachers (Nguyen, 2018; Li and Yao, 2022). Senior teachers report higher job satisfaction, greater classroom autonomy, more frequent evaluation and feedback, higher salaries, better internal professional development, and lower likelihood of leaving the profession (Li and Yao, 2022; Madigan and Kim, 2021). Teachers with higher academic qualifications, holding degrees in science or mathematics, those who have newborns within the first 3 years of teaching, or those residing farther from the school are more likely to leave the institution or the profession than their counterparts (Nguyen, 2018). Previous studies have found that teachers are less likely to leave their professional positions when they feel social value (Akiba et al., 2023; Arnold and Rahimi, 2025). However, there are significant regional differences in public and social perception of teachers (Arnold and Rahimi, 2025). And the current focus is mainly on Western backgrounds and limited attention to Asian backgrounds, so it is necessary to conduct more in-depth theoretical and empirical research on the impact of teacher mobility from different backgrounds.

Objectively speaking, the aforementioned factors influencing teacher retention do indeed exist. However, the impoverished conditions in rural areas cannot be fundamentally transformed within a short period, and certain regions will remain relatively underdeveloped. We must confront an objective reality: in relatively underdeveloped areas, salary and welfare levels themselves are inherently difficult to reach high standards. Furthermore, frequent teacher turnover exacerbates the training and financial burdens on schools and governments (Oke et al., 2016). Retaining rural teachers is both a critical and challenging task. Therefore, it is essential to comprehensively examine and analyze the factors affecting rural teacher retention. Although existing studies have proposed various dimensions of these influencing factors from multiple perspectives, the hierarchical structure among these factors remains unclear—specifically, which factors are most fundamental, and which are most immediate? Consensus has not yet been reached on this issue. Moreover, China’s vast rural territories and cultural diversity render such research particularly valuable as a reference. This study focuses on the rural teacher population, employing qualitative analysis to explore key factors affecting their retention. By constructing a theoretical model of influencing factors and an interpretative structural modeling (ISM) framework for rural teacher retention, this research aims to clarify the specific hierarchical structure of these influencing factors, thereby providing theoretical support and practical guidance for optimizing relevant policies, enhancing rural teacher retention rates, and improving their job satisfaction and wellbeing.

## 2 Literature review

In the analysis of rural teacher mobility and retention issues, certain scholars propose that the inquiry can be categorized into three major aspects: individual characteristic traits, school characteristics, and work-related attributes (Borman and Dowling, 2008). At the individual level, Most reasons for teacher turnover, including demographic variables, personal characteristics, teachers’ responses to their work, and wellbeing, are individual-level factors (Räsänen et al., 2020; Madigan and Kim, 2021). Generally, older teachers (compared to younger teachers), male teachers (compared to female teachers), and middle school teachers (compared to primary school teachers) exhibit higher intentions to leave (Li et al., 2019). Regarding personal characteristics, teachers with higher levels of intrinsic motivation typically demonstrate lower turnover intentions (Grant et al., 2019). In addition to demographic variables and personal traits, variables related to teachers’ responses to their work also significantly influence mobility intentions (Li and Yao, 2022). Work engagement, professional identity, and commitment serve as protective factors negatively associated with teacher turnover; burnout, in contrast, is positively associated with teacher attrition (den Brok et al., 2017; Tvedt et al., 2019). At the school level, factors such as remote geographical locations, inconvenient transportation facilities, isolation from social support systems and medical services, and underdeveloped economic conditions collectively contribute to teachers’ reluctance to work in rural areas (Echazarra and Radinger, 2019). And school leadership is strongly associated with teachers’ intent to remain at their schools; salary has a very weak relationship with overall retention rates but a significant relationship with retention rates within specific disciplines (Gundlach et al., 2024). Some studies have also indicated that teachers of mathematics, science, special education, and foreign languages are more likely to resign (Curran et al., 2019). At the work-related attributes, teachers in private schools, as well as those experiencing increased administrative workloads, heightened bureaucracy, and reduced administrative support, are associated with higher turnover rates (Gundlach et al., 2024). Moreover, teacher mobility is often closely related to the work atmosphere and teaching pressure (Zavelevsky and Lishchinsky, 2020). In addition to the three major categories of reasons mentioned above, a potential internal factor contributing to the attrition of rural teachers is the declining number of candidates choosing teaching as a profession compared to other industries (Frei et al., 2017).

Severe inequality in the allocation of educational resources between urban and rural areas in China. In 2020, there were 2,089,431 junior high school graduates in rural areas; however, only 777 academic high schools existed in these regions, admitting 346,370 students, resulting in a high school enrollment rate of merely 16.58% (Wu, 2022). In urban areas, there were 5,714,506 junior high school graduates, 7,414 academic high schools, and 4,317,496 students admitted, resulting in an enrollment rate of 75.55%, more than four times that of rural areas (Wu, 2022). Even more striking is that Beijing alone has 363 high schools, compared to only 353 in the four poor provinces of Hainan (142), Tibet (40), Qinghai (101), and Ningxia (70) (Ministry of Education of the People's Republic of China, 2024). At the same time, a large number of rural laborers frequently seek employment outside their home villages (Yiu and Yun, 2017), leading to a decline in rural population and student numbers, while the number of left-behind children has relatively increased (Xue et al., 2021). Research indicates that rural families generally possess weaker family capital, and parents are often absent. Furthermore, 37.2% of rural educators and frontline teachers believe that rural education policies provide greater or high attention to family education; 35.3% consider that these policies offer substantial or considerable attention; and 27.5% perceive that attention to family education is limited or insufficient (Xue et al., 2021). From the perspective of most rural teachers, the family education of these left-behind children is either inadequate or entirely absent; thus, teachers must exert significantly greater effort to achieve expected educational outcomes.

Efforts have been made worldwide to retain rural teachers. High-quality teachers are not only crucial for cultivating outstanding talents but also significantly influence regional stability and economic development. Many countries facing teacher shortages have adopted compensatory wage policies or financial incentives to attract and retain rural educators (Jiang and Yip, 2024). For example, the United States enacted the No Child Left Behind Act (NCLB) and the Every Student Succeeds Act (ESSA) (Swain et al., 2019); the United Kingdom launched the “Every Child Matters” initiative and the “Children’s Plan” (Hargreaves, 2009); India implemented Sarva Shiksha Abhiyan (National Education Movement) (Jan, 2017) and Rashtriya Madhyamik Shiksha Abhiyan (National Mission for Secondary Education) (Rashtriya Madhyamik Shiksha Abhiyan (RMSA), 2017). In China, the “Implementation Measures for the Rural Teacher Support Program (2015–2020)” (The State Council of the People's Republic of China, 2015) and the “Opinions of the Ministry of Education and Five Other Departments on Strengthening Rural Teacher Team Building in the New Era” (Ministry of Education of the People's Republic of China, 2020) were introduced. The Implementation Measures for the Rural Teacher Support Program (2015–2020) proposes broadening the sources of rural teachers, encouraging aspiring youth to dedicate themselves to rural education, and establishing smooth channels for college graduates and urban teachers to work in rural schools. It aims to gradually establish an incentive mechanism under which “the more one works in grassroots or harsher environments, the higher their status and benefits become” (The State Council of the People's Republic of China, 2015). The “Opinions of the Ministry of Education and Five Other Departments on Strengthening Rural Teacher Team Building in the New Era” relaxes the academic qualification requirements for title evaluation for primary and secondary school teachers who have long served in rural and remote, arduous areas (Ministry of Education of the People's Republic of China, 2020). Although these material incentives can to some extent effectively retain teachers (Manundu, 2023), the teachers would find it difficult to sustain long-term employment once such rewards are discontinued (Glazerman et al., 2006). Therefore, to retain rural teachers, it is essential to deeply understand what they truly desire. Simply increasing salary and benefits may attract teachers, but they may not be able to sustain a high-quality educational career in the long term.

This serves as a reminder that retaining rural teachers should not solely focus on material aspects; perhaps what they inwardly crave more is spiritual care. The equitable distribution of teaching resources serves as a moderating factor in teacher turnover (Borman and Dowling, 2008). Additionally, research has identified that the principal’s effectiveness in providing support, professional engagement, alignment between principals and teachers, alignment between teachers and students, and a high sense of achievement among students are critical factors in reducing turnover (Nguyen et al., 2020). Existing studies have examined the influencing factors of rural teacher mobility and retention from multiple perspectives. At the individual level, factors such as age, gender, academic level, intrinsic motivation, and work-related responses (e.g., work engagement, professional identity, and burnout) affect teachers’ intentions to move. At the school level, geographic location, transportation infrastructure, economic conditions, and school leadership influence teacher retention. Regarding job-related attributes, school type, administrative workload, work climate, and teaching pressure are associated with teacher mobility. These studies provide a theoretical foundation and research context for the present investigation. Although existing research offers a multidimensional perspective on influencing factors, the hierarchical structure among these factors remains unclear—there is no consensus on which factors are fundamental versus proximate. Furthermore, current research predominantly focuses on Western contexts with limited attention to Asian settings, particularly China; there is a lack of in-depth theoretical and empirical studies on teacher mobility across different cultural backgrounds. This study aims to address these gaps by conducting field investigations in rural China with the objective of identifying the hierarchical structure of factors influencing rural teachers.

## 3 Research design

This study focuses on teachers currently employed in rural areas, utilizing semi-structured interviews to gather data. Initially, the grounded theory approach is employed to construct a model of factors influencing the retention of rural teachers. Subsequently, the ISM (interpretive structural modeling) is applied to analyze the hierarchical relationships and interconnection pathways among these factors.

### 3.1 Research methodology

Grounded theory is a structured yet flexible research methodology, particularly suitable for situations where knowledge about a phenomenon is scarce. Its objective is to construct an explanatory theory (Chun Tie et al., 2019). Given the absence of a mature theoretical framework for understanding the factors influencing the retention of rural teachers and the evident limitations of existing theories in capturing the unique attributes of rural teachers in the specific context of China, this study employs the grounded theory approach.

The interpretive structural modeling (ISM) is a computer-based technique that constructs a framework of interrelationships among elements within a specific domain through the in-depth understanding of experts. This method is commonly employed to represent the mutual associations between various elements pertinent to a given problem (Shalamzari, 2023).

### 3.2 Data collection

This study employs purposive sampling and collects data through semi-structured interviews. Interviews with rural school teachers revealed that 3 years is a critical threshold for determining whether a teacher will stay. Most teachers who leave rural schools do so within the first 3 years of their assignment, and some young teachers even depart in the very first year. Moreover, a teaching supervisor noted, “It’s extremely difficult to cultivate a young, qualified teacher; it takes considerable time and effort before he truly develops teaching competence—typically two to 3 years.” Therefore, teachers who remain in rural schools for more than 3 years generally possess both the potential motivation to commit long-term to rural education and proven teaching experience. Such a teacher possesses the essential qualities of both “rural” and “teacher”—one who understands rural education and has some practical experience in it. So the interviewees are selected based on the following criteria: (1) having taught in rural areas for more than 3 years; (2) willingness to participate in the interview with clear and fluent language expression. Finally, a total of 33 teachers’ interview data were ultimately selected for analysis. The basic information of the interviewees is shown in Table 1.

**Table 1:** List of respondents.

**Number**	**Gender**	**Teaching segment**	**Teaching subject**	**Teaching experience**
1	Female	Grade 9	English	8 years
2	Female	Grade 4	Chinese	12 years
3	Female	Grade 6	English	11 years
4	Female	Grade 4	Chinese	26 years
5	Female	Grade 5	Math	5 years
6	Female	Grade 6	Chinese	25 years
7	Female	Grade 1	Chinese	27 years
8	Female	Grade 7	Politics	4 years
9	Male	Grade 8	Chemistry	5 years
10	Female	Grade 5	Chinese	18 years
11	Female	Grade 8	Math	13 years
12	Female	Grade 7	Chinese	28 years
13	Female	Grade 7	Math	7 years
14	Female	Grade 4	Math	4 years
15	Female	Grade 6	Chinese	15 years
16	Female	Grade 8	English	4 years
17	Male	Grade 8	P.E	6 years
18	Female	Grade 5	Chinese	23 years
19	Male	Grade 2	Math	4 years
20	Male	Grade 7	Geography	8 years
21	Female	Grade 7	History	11 years
22	Female	Grade 6	English	6 years
23	Female	Grade 9	History	5 years
24	Male	Grade 5	Chinese	4 years
25	Male	Grade 9	English	12 years
26	Female	Grade 8	Geography	16 years
27	Female	Grade 6	English	4 years
28	Female	Grade 8	History	20 years
29	Male	Grade 9	Physics	18 years
30	Female	Grade 5	Chinese	7 years
31	Male	Grade 8	Biology	8 years
32	Female	Grade 3	Music	4 years
33	Female	Grade 9	Chemistry	4 years

Based on the initial interview findings, the study designed an interview outline focusing on factors influencing the retention of rural teachers, primarily addressing four aspects: career choice and intention to stay, work environment and organizational support, salary and policy protection, and professional identity and emotional support. The detailed interview outline is provided in Appendix A. During the interview process, adjustments are made based on the interviewees’ understanding of the questions and their responses to ensure the authenticity of their thoughts and to guarantee the depth and comprehensiveness of the interview data. The research was approved by the Biomedical Research Ethics Committee of Hunan Normal University; with license number 2025 (955).

## 4 Data analysis and model building

### 4.1 Open coding

Open coding constitutes a component of the qualitative analysis process. Qualitative data, upon undergoing coding, facilitates researchers in categorizing and analyzing the data. Post-coding, qualitative data significantly enhances the potential for analysis and the formulation of new theories and concepts based on the generated codes. Open coding employs an inductive approach, mandating researchers to scrutinize the data with minimal preconceived notions. They should remain “open” to various possibilities of meaning within the data, thereby ensuring that the meanings encapsulated by the research project codes closely align with the original data’s intended expressions.

During the coding process, we established codes based on logical groupings of individual characteristics, school-related characteristics, and work-related attributes influencing the retention of rural teachers. Initial concepts representing individual characteristics included A5 Physical-condition, A7 Teaching-ability, and A27 Family-Development-Planning; initial concepts representing school characteristics included A1 Leadership-style, A6 Instructional-Evaluation-System, and A31 School-facilities; initial concepts reflecting job-related attributes included A3 Commuting-distance, A11 Job-restrictions, and A21 Teaching-pressure (Table 2). These initial concepts were then categorized, resulting in 17 sub-categories (Table 3).

**Table 2:** Conceptual extraction in open coding.

**Number**	**Initial concept**	**Number**	**Initial concept**
A1	Leadership style	A21	Teaching pressure
A2	Interpersonal relationship	A22	Policy benefits
A3	Commuting distance	A23	Salary
A4	Commuting difficulty	A24	Employment pressure
A5	Physical condition	A25	Parental involvement
A6	Instructional evaluation system	A26	Family culture
A7	Teaching ability	A27	Family development planning
A8	Value pursuit	A28	Student−teacher emotional bond
A9	Capacity improvement	A29	Student quality
A10	Love	A30	Number of students
A11	Job restrictions	A31	School facilities
A12	Policy restrictions	A32	Team atmosphere
A13	Management system	A33	Training system
A14	Social status	A34	Teaching resources
A15	Development opportunities	A35	Reward mechanism
A16	Self-improvement	A36	Accommodation
A17	Promotion mechanism	A37	Housing subsidies
A18	Educational mission	A38	School merger
A19	Team level	A39	Children’s education
A20	Teaching environment	A40	Humanistic care

**Table 3:** Categorization of open coding.

**Number**	**Sub-category**
B1 Teaching atmosphere	A1; A2; A19; A32
B2 Work stress	A5; A21; A24
B3 Teaching management system	A6; A16; A17; A33
B4 Teaching ability	A7; A9; A15
B5 Professionalism	A8; A10
B6 Policy	A11; A12; A13; A22; A35; A38
B7 Social status	A14
B8 Value pursuit	A18
B9 Wages and benefits	A23; A37
B10 Parental quality	A25
B11 Educational complex	A26
B12 Development plan	A27; A39
B13 Teacher−student emotions	A28
B14 Student quality	A29
B15 Teaching resources	A34
B16 Number of students	A30
B17 School facilities and humanistic care	A3; A4; A20; A31; A36; A40

### 4.2 Axial coding

Based on open coding, the study explores the connections between various category types, repeatedly compares and analyzes the conceptual connotations and interrelationships of sub-categories, thereby deriving main categories. Through axial coding, the research refines five categories, including “School Environment and Resource Conditions, “Management Systems and Policy Environment,” as detailed in Table 4.

**Table 4:** Results of axial coding.

**Main category**	**Sub-category**	**Category connotation**
C1 Teacher Qualifications and Health	B2 Work stress	Professional competence and physical health of teachers
	B4 Teaching ability	
	B5 Professionalism	
	B8 Value pursuit	
	B11 Educational complex	
	B12 Development plan	
C2 School Environment	B1 Teaching atmosphere	School infrastructure
	B15 Teaching resources	
	B17 School facilities and humanistic care	
C3 Policy and Institutional Frameworks	B3 Teaching management system	Teacher management system and promotion pathways
	B6 Policy	
C4 Job Compensation and Career Prospects	B7 Social status	Wages, benefits, and allowances
	B9 Wages and benefits	
C5 Student and Family Influencing	B10 Parental quality	Student quality, parental involvement, and the teacher’s own family planning.
	B13 Teacher−student emotions	
	B14 Student quality	
	B16 Number of students	

### 4.3 Selective coding

Selective coding, as the final step in grounded theory coding, constitutes a pivotal component of any grounded theory analysis, enabling researchers to derive key insights from their research. This process involves the selection of a core variable or concept from the existing categories established during the axial coding phase. In this study, five major domains influencing rural teachers’ retention were identified: C1 Teacher Qualifications and Health, C2 School Environment, C3 Policy and Institutional Frameworks, C4 Job Compensation and Career Prospects, and C5 Student and Family Influencing. Among them, C1 Teacher Qualifications and Health are categorized as individual characteristic influencing factors; C2 School Environment and C3 Policy and Institutional Factors fall under school-specific influencing factors; whereas C4 Job Compensation and Career Prospects and C5 Student and Family Influencing are classified as work-related attribute factors.

**Figure 1:**
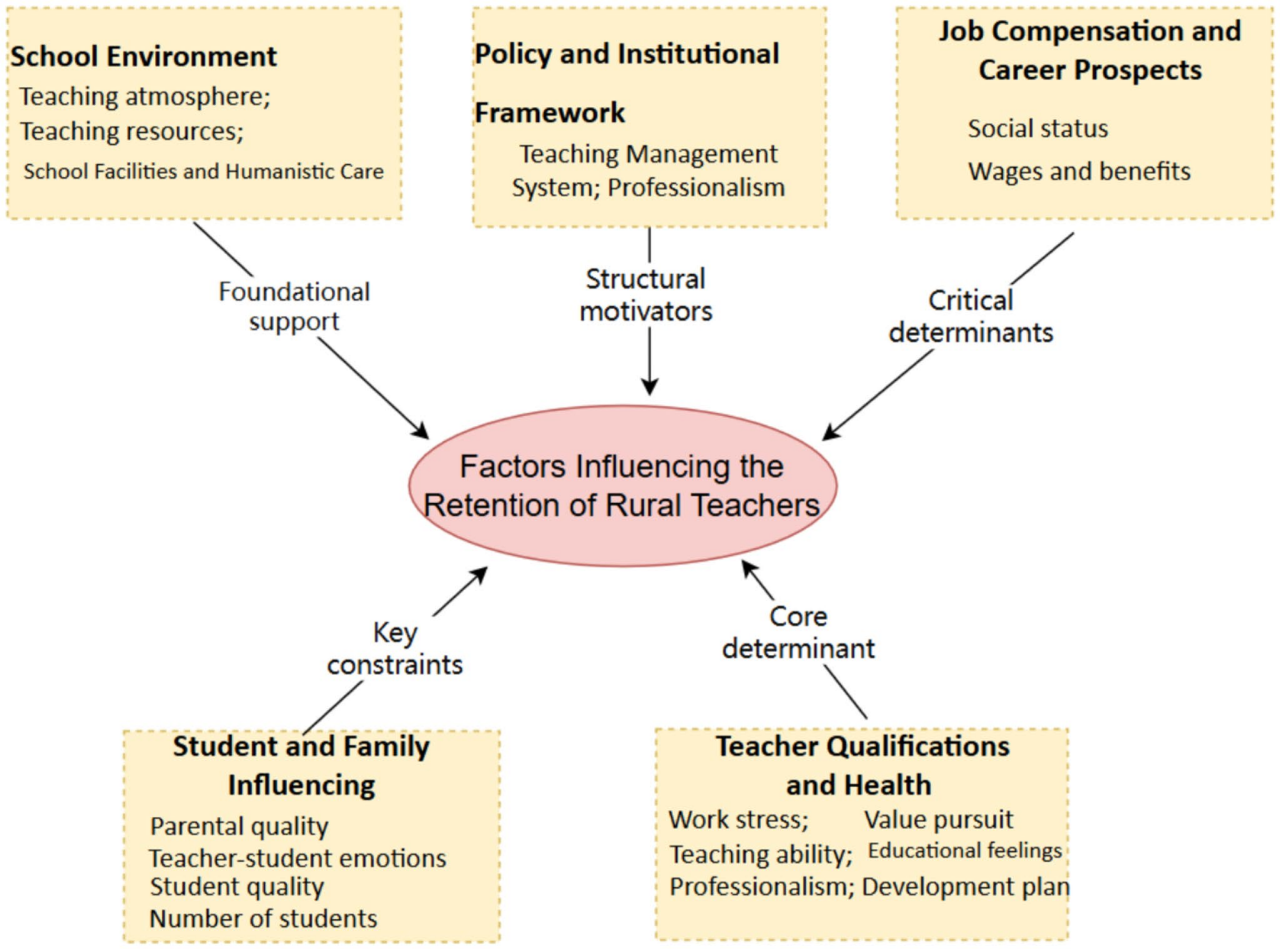
Theoretical model of factors influencing the retention of rural teachers.

Based on direct quotations from interview texts (e.g., “salaries in rural areas are too low to retain excellent teachers”; “school consolidations might affect student enrollment, thereby impacting teaching outcomes and teachers’ professional satisfaction”) and recurrent themes regarding material foundations and policy perceptions, a causal retrodiction method was employed to construct a relational network. Specifically: (1) Teacher Qualifications and Health constitute the core determinant. Teachers’ professional competencies, physical or mental wellbeing, and career development plans fundamentally anchor their long-term commitment to rural education; (2) The School Environment provides foundational support by offering essential teaching resources, a conducive atmosphere, and humanistic care, creating a sustainable working foundation that indirectly stabilizes retention; (3) Policy and Institutional Frameworks act as structural motivators. Through teaching management systems, professional evaluation standards, and incentives for rural service, these policies exert institutional influence to encourage retention, albeit indirectly via administrative mechanisms; (4) Job Compensation and Career Prospects serve as critical determinants that directly influence retention decisions. Competitive salaries, social status, and clear promotion pathways are pivotal in sustaining teachers’ economic security and professional motivation; (5) Student and Family Influencing imposes key constraints. Factors such as declining student enrollment, parental indifference, or strained teacher-student-parent relationships create tangible barriers, increasingly challenging teachers’ sense of efficacy and belonging in rural settings. Through multidimensional validation, a theoretical model integrating these five interrelated dimensions was developed (Figure 1).

To ensure the adequacy and completeness of theoretical construction, the two coders coded the two reserved interview transcripts, verifying through lateral comparison that no new concepts were generated, implying that the existing categories and relationships comprehensively encompass all concepts, thereby indicating that the coding results have reached theoretical saturation.

## 5 Interpretive structural modeling

This study employs the interpretive structural modeling (ISM) approach to delve into the intrinsic logical relationships among the factors influencing rural teachers’ retention, as derived from Grounded Theory analysis. A questionnaire (see Appendix B) survey was conducted among 15 leaders engaged in rural education and experts in rural education research, who were asked to provide fuzzy ratings on the pairwise relationships between the influencing factors. The selection of these leaders and experts was carefully considered: (1) the selected rural school teacher leaders are all supervisors of teaching staff within their respective schools, concurrently engaged in rural teaching duties and possessing extensive experience in managing teaching teams; (2) the experts were chosen from professors and scholars at normal universities specializing in rural education research, who hold profound insights into rural education. A total of 15 questionnaires were distributed, with 1 invalid questionnaire excluded, resulting in 14 valid responses for the final analysis.

### 5.1 Adjacency matrix construction

The statistical results of leaders and experts evaluations yield the fuzzy adjacency matrix F. Subsequently, the correlation strength matrix B is computed using the provided formula ( $b_{\mathrm{ij}}=\frac{f_{\mathrm{ij}}}{f_{i}+f_{j}-f_{\mathrm{ij}}}$ ), and the adjacency matrix A ( $a={\left[a_{\mathrm{ij}}\right]}_{n*n}$ ) is derived from the correlation strength matrix B ( $B={\left[b_{\mathrm{ij}}\right]}_{n*n}$ ), as detailed in Table 5.

**Table 5:** Adjacency matrix A.

**Factor**	**B1**	**B2**	**B3**	**B4**	**B5**	**B6**	**B7**	**B8**	**B9**	**B10**	**B11**	**B12**	**B13**	**B14**	**B15**	**B16**	**B17**
B1	0	0	0	0	0	0	0	0	0	0	0	0	0	0	1	0	0
B2	1	0	1	1	0	0	0	0	0	0	0	0	0	0	0	1	1
B3	0	0	1	0	0	1	0	0	0	0	0	0	0	0	0	0	0
B4	0	0	1	0	0	1	0	0	0	0	0	0	0	0	1	0	0
B5	0	0	1	1	0	1	0	0	1	0	0	0	0	0	0	0	0
B6	0	0	0	0	0	0	0	0	0	0	0	0	0	0	0	0	0
B7	0	0	0	0	1	1	0	0	0	0	0	0	0	0	0	0	0
B8	1	0	1	0	1	0	1	0	0	1	1	0	1	1	1	1	1
B9	0	0	1	1	0	1	0	0	0	0	0	0	0	0	0	0	0
B10	0	0	0	1	0	0	0	0	0	0	0	0	0	0	0	0	0
B11	0	1	0	0	0	0	0	0	0	0	0	0	0	0	0	0	0
B12	0	1	1	0	1	0	0	1	1	1	1	0	1	1	1	1	0
B13	1	0	0	0	0	0	0	0	0	0	0	0	0	0	0	1	1
B14	1	0	0	0	0	1	0	0	0	0	0	0	0	0	1	1	1
B15	0	0	0	0	0	1	0	0	0	0	0	0	0	0	0	0	0
B16	1	0	0	0	0	1	0	0	0	0	0	0	0	0	0	0	1
B17	0	0	0	0	0	1	0	0	0	0	0	0	0	0	0	0	0

### 5.2 Construction of reachability matrix

The reachability matrix serves to further elucidate the indirect connections and transmission pathways among influencing factors. By integrating the Boolean Algebra Laws, the adjacency matrix A is summed with the identity matrix I, followed by successive self-multiplications until the equality of two consecutive power matrices is achieved, at which point the computation ceases, yielding the reachability matrix M (as depicted in Table 6).

**Table 6:** Reachability matrix M.

**Factor**	**B1**	**B2**	**B3**	**B4**	**B5**	**B6**	**B7**	**B8**	**B9**	**B10**	**B11**	**B12**	**B13**	**B14**	**B15**	**B16**	**B17**
B1	1	0	0	0	0	1	0	0	0	0	0	0	0	0	1	0	1
B2	1	1	1	1	0	1	0	0	0	0	0	0	0	0	1	1	1
B3	0	0	1	0	0	1	0	0	0	0	0	0	0	0	0	0	0
B4	0	0	1	1	0	1	0	0	0	0	0	0	0	0	1	0	0
B5	0	0	1	1	1	1	0	0	1	0	0	0	0	0	1	0	1
B6	0	0	0	0	0	1	0	0	0	0	0	0	0	0	0	0	0
B7	0	0	1	1	1	1	1	0	1	0	0	0	0	0	1	0	1
B8	1	1	1	1	1	1	1	1	1	1	1	0	1	1	1	1	1
B9	0	0	1	1	0	1	0	0	1	0	0	0	0	0	1	0	1
B10	0	0	1	1	0	1	0	0	0	1	0	0	0	0	1	0	0
B11	1	1	1	1	0	1	0	0	0	0	1	0	0	0	1	1	1
B12	1	1	1	1	1	1	1	1	1	1	1	1	1	1	1	1	1
B13	1	0	0	0	0	1	0	0	0	0	0	0	1	0	1	1	1
B14	1	0	0	0	0	1	0	0	0	0	0	0	0	1	1	1	1
B15	0	0	0	0	0	1	0	0	0	0	0	0	0	0	1	0	0
B16	1	0	0	0	0	1	0	0	0	0	0	0	0	0	1	1	1
B17	0	0	0	0	0	1	0	0	0	0	0	0	0	0	0	0	1

### 5.3 Hierarchical relationship of decomposition elements

Based on the aforementioned reachability matrix M, the reachability set R (B_i_), antecedent set A (B_i_), and the intersection of the two, R (Bi)∩A (B_i_), are determined for the influencing factors of rural teacher retention. Based on the formula, the hierarchical structure of elements is delineated, with the exploration of new highest-level elements conducted sequentially. Each identified highest-level element is subsequently removed from M until the final layer of elements is delineated. The hierarchical decomposition results of factors influencing rural teacher retention are thereby obtained (see Table 7).

**Table 7:** Reachable set, antecedent set, and intersection.

**i**	**R(Bi)**	**A(Bi)**	**R(Bi)∩A(Bi)**
B1	1,6,15,17	1,2,8,11,12,13,14,16	1
B2	1,2,3,4,6,15,16,17	2,8,11,12	2
B3	3,6	2,3,4,5,7,8,9,10,11,12	3
B4	3,4,6,15	2,4,5,7,8,9,10,11,12	4
B5	3,4,5,6,9,15,17	5,7,8,12	5
B6	6	1,2,3,4,5,6,7,8,9,10,11,12,13,14,15,16,17	6
B7	3,4,5,6,7,9,15,17	7,8,12	7
B8	1,2,3,4,5,6,7,8,9,10,11,13,14,15,16,17	8,12	8
B9	3,4,6,9,15,17	5,7,8,9,12	9
B10	3,4,6,10,15	8,10,12	10
B11	1,2,3,4,6,11,15,16,17	8,11,12	11
B12	1,2,3,4,5,6,7,8,9,10,11,12,13,14,15,16,17	12	12
B13	1,6,13,15,16,17	8,12,13	13
B14	1,6,14,15,16,17	8,12,14	14
B15	6,15	1,2,4,5,7,8,9,10,11,12,13,14,15,16	15
B16	1,6,15,16,17	2,8,11,12,13,14,16	16
B17	6,17	1,2,5,7,8,9,11,12,13,14,16,17	17

### 5.4 Construction of interpretative structural modeling

**Figure 2:**
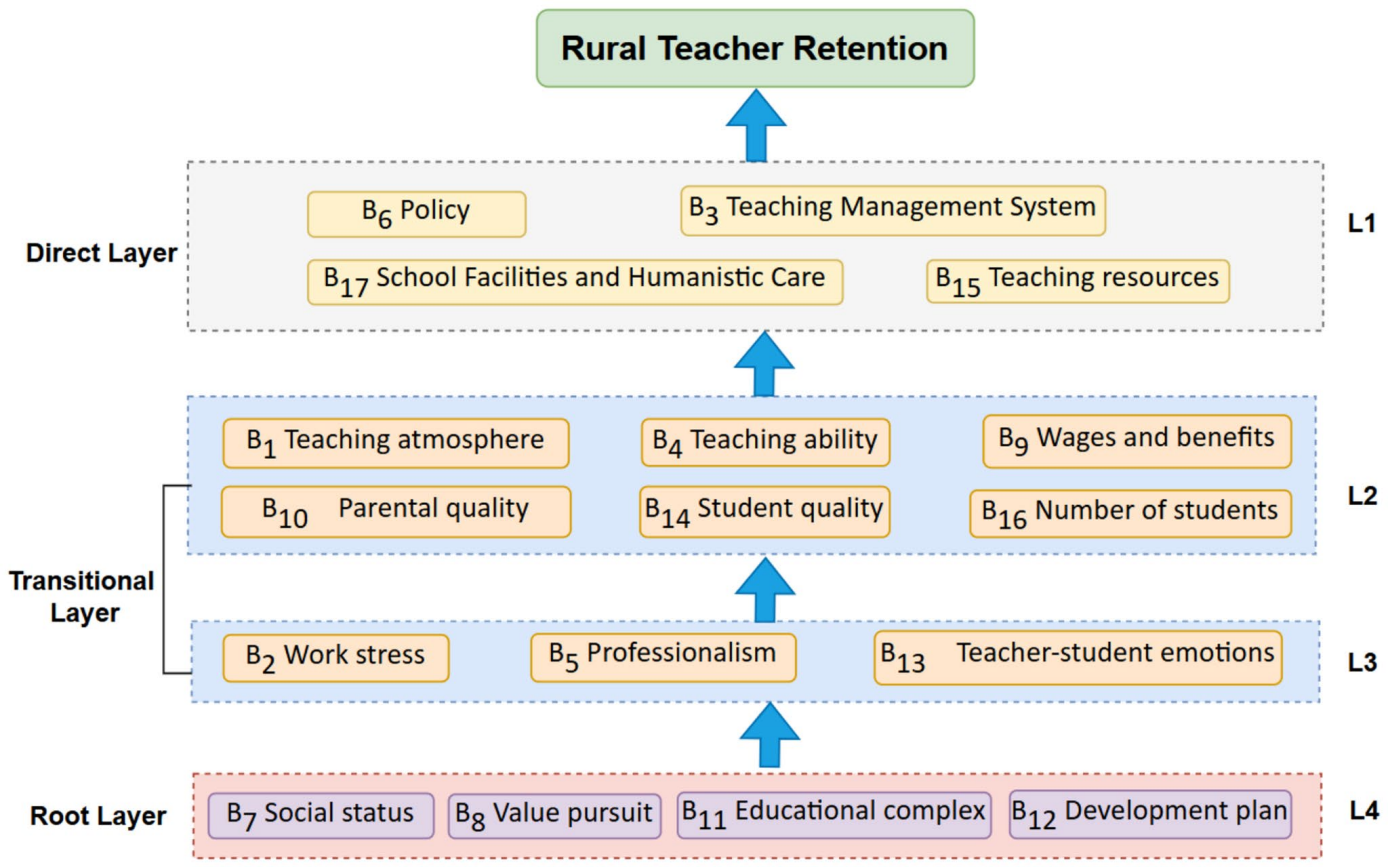
Explanatory structural modeling of factors influencing the retention of rural teachers.

Based on the aforementioned categorization results, an interpretative structural modeling (ISM) of the influencing factors on rural teachers’ retention is constructed, as illustrated in Figure 2.

The figure constructs a multi-level influencing factor model to analyze the complex driving mechanisms of rural teacher retention. The model is divided into four hierarchical levels, exerting influence progressively from the bottom to the top, revealing the underlying logic behind rural teachers’ retention decisions:

Direct Layer (L1): Composed of observable explicit factors, including policy (B6), teaching management system (B3), school facilities and humanistic care (B17), and teaching resources (B15). These factors directly influence teachers’ work experience: policy support (e.g., staffing security) and teaching management systems (e.g., evaluation mechanisms) provide institutional safeguards; school facilities and humanistic care enhance the working environment; and teaching resources meet the professional development needs related to teaching competence (Table 8).

**Table 8:** Hierarchical decomposition.

**Level**	**Factor**
L1	B6 Policy, B3 Teaching management system, B15 Teaching resources, B17 School facilities and humanistic care
L2	B1 Teaching atmosphere, B4 Teaching ability, B9 Wages and benefits, B10 Parental quality, B16 Number of students, B14 Student quality
L3	B2 Work stress, B5 Professionalism, B13 Teacher−student emotions
L4	B7 Social status, B11 Educational complex, B8 Value pursuit, B12 Development plan

Transitional Layer (L2, L3): Acting as an intermediary transformation function, it includes work stress (B2), professionalism (B5), teacher−student emotions (B13), wages and benefits (B9), and number of students (B16). Work pressure reflects the task load and psychological stress experienced by rural teachers; professional commitment represents teachers’ pursuit of professional development; teacher−student emotional connection emphasizes emotional fulfillment derived from interactions with students. This layer transforms the abstract motivations from the foundational level into concrete behavioral orientations—for instance, professional commitment may alleviate work pressure, while teacher−student emotional connection can strengthen occupational belongingness; salary and benefits satisfy economic needs, and student enrollment influences teaching workload and sense of accomplishment.

Root Layer (L4): As foundational driving factors, this layer includes social status (B7), value pursuit (B8), educational complex (B11), and development plan (B12). These factors reflect teachers’ intrinsic motivations: social status influences occupational identity, value pursuit determines their level of commitment to rural education, educational sentiment embodies personal emotional attachment to teaching, and development planning relates to expectations regarding career prospects. These deep-seated factors indirectly influence retention intention through the transitional layer.

## 6 Findings

According to that model, value pursuit at the foundational layer is transformed into teaching motivation through the transitional layer’s professionalism, ultimately influencing dependence on policies and resources at the immediate layer. The environment may play a moderating role. Material conditions and institutional design at the immediate layer can inversely alleviate work pressure in the transitional layer, thereby establishing a dynamic equilibrium. Furthermore, wages and benefits (B9), as an element of the transitional layer, are subject to policy (B6) while also resonating with social status at the foundational layer (B7), reflecting dual drivers of economic needs and social recognition. In summary, rural teachers’ retention can be understood as an outcome of interwoven internal and external factors. Deep-seated value identification must be translated into retention motivation through opportunities for professional development and emotional connection, while material security and institutional support form the practical foundation sustaining retention intentions. Policy interventions should emphasize multi-level coordination: for instance, enhancing teachers’ social status to strengthen occupational appeal, while simultaneously optimizing compensation and welfare systems to meet concrete needs, thus establishing a sustainable mechanism for teacher retention.

From the perspective of individual characteristic traits, female teachers have become the mainstay of rural education, with their retention primarily influenced by family-related factors. “I feel it is quite good to stay and teach in the countryside. Although it is a bit farther from home, I actually spend more time at home with my children each day. Schools near the city center do not finish until after 6 p.m., but here at our school, we can leave early as long as there are no teaching duties.” Male teachers, by contrast, often leave rural areas due to family responsibilities. Unmarried male teachers indicate they are highly likely to abandon rural education for family reasons. “Whether I stay in the countryside depends on my future plans and life outcomes. If I start a family or have children, I might have to reconsider my current job due to economic or educational concerns.” Therefore, teachers from stable families are more inclined toward long-term retention, while those owning private vehicles—benefiting from convenient commuting—are more adaptable to rural life. “My colleagues and I both have cars; we can drive home together and chat along the way—we are back home before we know it. So I do not see anything negative about staying here.” Furthermore, retained teachers generally possess a strong sense of educational mission and regard their work as a pathway to achieving personal fulfillment—particularly hoping that students can transform their lives through education. “I myself come from a rural background; I hope rural children can study well and eventually leave the countryside. This has always been my original motivation in education, and it remains unchanged today.”

From the perspective of school characteristics, Chinese and mathematics teachers have become the core pillars of rural schools, whereas teachers in subjects such as music, physical education, and art experience extremely high turnover rates. Schools allocate limited resources to non-core subject teachers and do not prioritize their professional development or training. As one physical education teacher remarked: “I feel no sense of accomplishment working in a rural school. The sports facilities are severely lacking, preventing me from conducting normal instruction. Moreover, school administrators do not value my work, and my salary is lower than that of core subject teachers.” Meanwhile, school administrators argue that training young teachers requires mobilizing the entire school’s resources; however, these teachers often leave shortly after completing training, resulting in low return on investment. Consequently, schools have gradually reduced their resource allocation. This imbalance in resource distribution combined with inadequate school facilities further exacerbates the structural imbalance between core and non-core subject teachers, creating a vicious cycle.

From the perspective of work-related attributes, the government has guided teachers toward rural areas through contractual policies, thereby retaining some educators to a certain extent. “I never considered leaving the countryside because I signed a contract; if I were to breach it, I would have to pay a penalty fee and my name would be recorded in a credit file.” Nevertheless, teachers who wish to leave rural areas are willing to pay the penalty fee in order to resign. The relatively manageable work pressure and simple interpersonal relationships in rural teaching positions also contribute significantly to teachers’ decisions to remain. However, low salaries and inadequate benefits often discourage teachers with economic needs from staying—this is particularly evident among male teachers.

## 7 Discussion

This study focuses on the influencing factors and their interrelated pathways affecting the retention of rural teachers in China, aiming to reveal the complex factors and hierarchical structure underlying rural teacher retention. Employing grounded theory and interpretive structural modeling (ISM), this research conducts an in-depth analysis of semi-structured interview data from 33 rural teachers to develop a theoretical model of factors influencing rural teacher retention. Findings indicate that gender differences, subject-specific characteristics, policy drivers, and value orientations significantly impact teacher retention, with complex hierarchical relationships among these factors. The discussion section first reviews the research questions and methodology, emphasizing key findings. It then compares the results with existing literature to analyze consistencies and discrepancies and their underlying causes. Subsequently, unexpected findings are discussed, followed by an explanation of how the results contribute to existing theoretical models. Finally, limitations of the study are acknowledged.

The findings of this study are consistent with numerous existing literatures. The study identifies salary compensation, working conditions, and social welfare as critical factors influencing teacher retention, a finding that aligns with Nguyen and Springer (2021). Furthermore, this study reveals differential impacts of these factors across teachers of different genders and subject disciplines, echoing Hudson and Hudson (2019) findings on the challenges of teacher retention in rural and remote areas. However, certain results diverge from prior literature. For instance, contrary to Sutcher et al. (2019) conclusion in the U.S. context—that increasing salaries significantly reduces teacher turnover—this study finds that merely raising salaries does not universally resolve retention issues among rural teachers, particularly for male teachers. Economic pressure is only one contributing factor; family responsibilities and career development prospects are equally significant. This discrepancy may stem from differences in national contexts, cultural backgrounds, and the unique characteristics of rural educational environments. An unexpected finding of this study is the pronounced influence of family stability on female teachers’ retention. While prior literature has acknowledged the role of family-related factors in teachers’ career choices, this study provides deeper insight into how family stability promotes long-term retention among female teachers by alleviating personal concerns, enhancing professional identity, and strengthening a sense of belonging. Additionally, the study identifies a strong sense of “educational mission” among rural teachers as a key intrinsic motivation for retention—an insight that offers new perspectives for enhancing the occupational appeal and retention rates of rural educators.

By constructing a hierarchical structural model of factors influencing rural teachers’ retention, this study clarifies the hierarchical relationships and pathways among various influencing factors, revealing the interactive effects of material conditions and psychological needs in teachers’ decisions to remain. The findings indicate that social status, value orientation, and career development plans constitute deep-level factors affecting rural teachers’ willingness to stay; meanwhile, policies, teaching facilities, and resources serve as direct factors shaping school environment and working conditions. Therefore, targeted policies should adopt long-term strategies centered on promoting the spirit of educational leadership, a sense of mission, and professional responsibility. At the same time, immediate measures such as flexible utilization of policy instruments and management systems should be employed to attract teachers. Furthermore, transitional factors such as teaching atmosphere and salary benefits should be effectively leveraged to flexibly adjust management strategies across different stages of educational development, thereby ensuring stability within the rural teacher workforce. This model not only enriches theoretical understanding of rural teacher retention but also provides policymakers with a more comprehensive theoretical foundation for designing more precise and effective intervention strategies.

### 7.1 Contributions

The value of this study is that it not only systematically sorts out the four major influencing factors including individual characteristics, school environment, policy system and career prospects, but also innovatively identifies social status, professional identity and educational mission as the core forces driving rural teachers’ retention, thus surpassing the single focus on material conditions in previous studies. The marginal contribution of this study is mainly reflected in the theoretical level, which provides a multi-dimensional analytical framework, combines deep cultural and psychological factors with external material conditions, deepens the understanding of rural teacher retention mechanism, and provides a new theoretical perspective and analytical tool for subsequent research. At the same time, the study also provides a valuable reference for policymakers, emphasizing that while improving the treatment of rural teachers, it is necessary to pay more attention to the improvement of their social status, the enhancement of professional identity and the stimulation of the sense of educational mission, so as to build a more comprehensive and sustainable incentive mechanism for teacher retention.

### 7.2 Limitations

Although this study has achieved significant findings in revealing the influencing factors and their interrelated pathways regarding rural teachers’ retention, several limitations remain. First, the sample size is limited, which may introduce sampling bias; future research should expand its geographical coverage to enhance the generalizability of the results. Second, this study primarily employs qualitative methods; future research could integrate quantitative approaches to further validate and complement the current findings.

## 8 Countermeasures and implications

Previous strategies addressing the retention of rural teachers have generally been highly universalistic, emphasizing the role of rural infrastructure and salary benefits. However, survey findings indicate that different demographic groups are influenced by distinct factors in their decisions regarding teacher retention. The interpretive structural modeling results reveal that policies and management systems constitute foundational-level factors directly affecting school environments and working conditions, whereas career prospects, social status, and values represent higher-order factors that profoundly influence long-term intentions to remain in teaching. Therefore, this study targets the root-level influencing factors and develops tailored solutions according to the distinct characteristics of various groups, aiming to address challenges that have not been sufficiently explored or adequately handled in prior research.

### 8.1 Differentiated retention strategies for teachers of different genders

Implementing family-friendly policies for female teachers, such as flexible working hours, extended maternity and breastfeeding leave, helps them better balance family and professional responsibilities. Recognizing the current underrepresentation of women in leadership positions, there is a call to provide female teachers with greater opportunities for career advancement and leadership roles, encouraging them to pursue higher achievements throughout their careers and thereby strengthening their professional identity and intention to remain in the profession.

To address the issue of male teachers leaving due to economic pressure, schools should offer more competitive salaries and benefits, while providing clear career development pathways to help these educators envision long-term prospects in rural education. Specifically, schools can establish more challenging positions with correspondingly higher compensation to attract capable but financially strained male teachers. Regarding marriage, organizing matchmaking events and offering marital and family counseling can assist unmarried male teachers in resolving personal relationship issues, thereby enhancing their willingness to remain in their positions.

### 8.2 Retention strategies for teachers in specific academic disciplines

Core subject teachers adopt a stable strategy. Special development funds are provided for core subject teachers—such as those in Chinese and Math—to support their teaching research and innovative practices, thereby enhancing their professional competence and instructional capabilities. Free psychological counseling and stress management training are offered to core subject teachers, along with regular team-building activities to alleviate occupational anxiety. Through documentaries, public service advertisements, and social media campaigns, their professional pride and intention to remain in the profession are strengthened.

Subject-specific teachers adopt attraction-oriented strategies. In response to the high turnover rate among subject-specific teachers—such as physical education, music, and art teachers—expanding their career development pathways can help enhance their professional engagement and retention. This includes providing greater opportunities for artistic creation and performance, organizing interdisciplinary teaching collaborations, and fostering a sense of professional fulfillment. Additionally, improving their salary and benefits, as well as increasing their recognition within schools and society, can enhance the attractiveness of their profession and strengthen their intention to remain in the field.

### 8.3 Retention strategies for policy-driven teachers

Policy-driven teachers typically enter rural education due to government policy incentives, but often choose to leave after their contract period ends. Therefore, more favorable renewal terms should be offered to those willing to continue teaching in rural areas after their contracts expire, thereby establishing sustained policy appeal. Given that some teachers lack sufficient consideration when initially signing up for such policies, transparency during policy implementation becomes essential. In the future, teachers should be clearly informed about policy content, implementation standards, and expected outcomes to strengthen their trust in and sense of belonging to the policy. Furthermore, the government should regularly evaluate the effectiveness of policy implementation and collect feedback from teachers and schools to provide a basis for ongoing policy optimization.

### 8.4 Retention strategies for teachers in stable and happy families

Although teachers who currently enjoy a happy and stable family life are willing to stay long-term in rural schools, corresponding support strategies are still needed to reinforce their intention to remain. Implementing family care programs for such teachers—such as educational support for their children, employment assistance for spouses, and elderly care services—can alleviate their concerns about family matters. Secondly, regular family day events can be organized to invite teachers’ family members to participate, thereby strengthening the connection between families and schools and enhancing teachers’ sense of familial belonging and professional satisfaction.

### 8.5 Implications

This study conducts an in-depth analysis of rural teacher retention in China, offering significant implications for policy formulation and educational practice. From a policy perspective, the research demonstrates that relying solely on material incentives, such as salary increases, is insufficient to comprehensively address the challenges of rural teacher retention. It underscores the importance of enhancing teachers’ social status, strengthening professional identity, and fostering a sense of educational mission. Consequently, policymakers should design integrated incentive systems that go beyond economic compensation, incorporating policy guidance and public awareness campaigns to elevate teachers’ professional prestige, while also expanding opportunities for career advancement and promotion pathways for rural educators. In educational practice, school administrators should prioritize improving working conditions in rural schools by providing adequate teaching resources and infrastructural support. At the same time, attention must be paid to teachers’ psychological wellbeing and professional development needs through measures such as family-friendly policies, reduction of non-teaching burdens, enhanced team collaboration, and strengthened psychological support systems—thereby increasing job satisfaction and a sense of belonging among teachers. Furthermore, differentiated retention strategies should be developed based on gender and subject-specific characteristics to better meet the distinct needs of various teacher groups. Ultimately, this approach aims to establish a comprehensive, multi-tiered support system for rural teacher retention.

## 9 Conclusion

This study comprehensively examines the influencing factors and associated pathways affecting the retention of rural teachers in China, employing grounded theory and interpretive structural modeling (ISM) to analyze survey data from 33 rural teachers. The findings indicate that underlying factors influencing teacher retention remain cultural and intangible, rather than objective material conditions. Therefore, simply increasing salary benefits is not universally applicable to all rural teachers. Furthermore, the factors affecting teacher retention exhibit group-specific characteristics. The innovation of this study lies in its multidimensional analytical framework, which integrates individual, institutional, and policy-level factors to construct a hierarchical model of rural teacher retention. By identifying “social status,” “value pursuit,” and “educational mission” as fundamental driving forces, this study transcends a narrow focus on material conditions and offers a more profound understanding. However, limitations remain, including potential sampling bias. Future research should expand geographical coverage to enhance the generalizability of findings. Additionally, in-depth evaluation of specific interventions—such as family-friendly policies and professional development programs—could provide policymakers with more actionable insights. Overall, this study lays a foundation for further exploration of the complex dynamics underlying rural teacher retention and underscores the necessity of comprehensive strategies that address both material needs and psychological wellbeing simultaneously.

## Data Availability

The raw data supporting the conclusions of this article will be made available by the authors, without undue reservation.
