# Supplementary material for: Research on influencing factors and correlation pathways of rural teachers’ retention in China
Source: Front Psychol. 2026 Jan 12;16:1728628. doi: 10.3389/fpsyg.2025.1728628 (PMC12833959; doi:10.3389/fpsyg.2025.1728628)
Supplement: Supplementary file 1 [file Data_Sheet_1.pdf]

Appendix A Excerpt From the Interview

Interviewee 1

Q1: What was your original motivation for becoming a rural teacher? Has it changed over time?

A1: To realize my personal value and do my best to nurture every student I can. I've stayed true to that original intention—serving the people and never forgetting why I started.

Q2: Have you ever thought about leaving the rural school? If so, what were the specific reasons? What ultimately made you decide to stay?

A2: Yes, I've had thoughts about leaving. The main reasons were parents not being supportive enough and students not having good habits yet. The reasons I decided to stay were multifaceted — both personal and environmental. Personally, I've been putting in extra effort to read professional books and improve my skills, hoping to pass the promotion exam soon and move to a city school.

Q3: Do you think the current school's working environment—like hardware facilities and teaching resources—meets teaching needs?

What areas need improvement?

A3: Overall, 90% of it meets our needs. The main improvements are the new playground and the big upgrade in the school's appearance. The leadership team is young and really motivated to improve the school and raise teaching standards. Since we're located at the urban-rural fringe and are the closest school to town, transportation is convenient. Plus, our teachers are mostly young, so the whole school atmosphere—management and teaching alike—is becoming more energetic. The younger staff bring fresh ideas, energy, and strong execution skills—definitely a big plus. Honestly, being around them makes me feel younger too.

Q4: How's your relationship with your colleagues and school leaders? Does it affect your motivation at work?

A4: No burnout—just address issues as they come up, always see myself improving. I've also noticed the school leadership has been fully supportive and cooperative with my work, which makes me feel my efforts truly matter here.

Q5: Are you satisfied with your current salary and benefits? Do you think pay is a key factor affecting retention?

A5: First of all, I'm a pretty down-to-earth teacher—no fancy stuff, but I still believe in maintaining a decent quality of life. That said, earning extra income by moving to the city isn't why I came here. What really drew me in was the better work environment, better students, and more supportive parents. I feel my educational impact, professional achievements, and sense of accomplishment grow much more here. Of course, in the countryside, salary is tied to your job title. Since I started late, I haven't gotten my title yet. If I did, my pay would definitely go up. That'd definitely

help improve my family's finances and standard of living. But honestly, a person's quality of life isn't just about money—it's also about mental fulfillment. When you're in an environment where you're constantly growing as a teacher and your students are growing too, that inner joy and sense of achievement can't be bought with cash. For me, seeing real progress in my students—knowing they've been shaped by my teaching—that's what truly matters most. Honestly, that kind of success means way more to me than any paycheck ever could.

Q6: Are you aware of and have you benefited from the special policy for rural teachers (like subsidies, housing security)? Were there any issues during the implementation?
A6: Yes, I did. I won't say it's always on time, but at least the payments were guaranteed and actually made. They're not sent out monthly on schedule, but at least we always got our basic salary.

Q7: What other forms of support do you think the government or schools could provide to make rural teaching more attractive?
A7: To keep rural teachers, it's not just about improving physical facilities. Since our school is relatively close to town, many teachers have short commutes and find it very convenient to get to work. The real issue is whether rural teachers can stay on the job long-term, which depends heavily on the teaching environment. Many teachers are discouraged by parents and students — feeling they can't achieve personal fulfillment and find it hard to teach and manage students. When parents and students don't cooperate, young teachers gradually lose motivation and start thinking about quitting or just going with the flow.

Interviewee 2:
Q1: What was your initial motivation for becoming a rural teacher? Has it changed over time?
A1: To contribute my part to rural education. My original motivation hasn't changed.

Q2: How do you see your future career development? Do you have plans to stay in the countryside long-term?
A2: If there are still rural schools left, I'd probably stay in the countryside for a long time.

Q3: Do you think the current school working environment (like hardware facilities, teaching resources) meets teaching needs?
What areas need improvement?
A3: Most of the time it's sufficient, but I think the school could improve some teaching facilities and sports equipment. Also, teachers' housing could be better too.

Q4: Do you recognize the professional value of rural teachers? How does this recognition affect your willingness to stay?

A4: Definitely. It makes you feel more valued.

Q5: Does your emotional connection with students affect your commitment to rural education? Can you share a specific example?
A5: Definitely. It's kind of satisfying when they come back to visit you after you've taught them.

Q6: If you leave the countryside in the future, what do you think would be the most likely reason?
A6: Being too far from home.

Interviewee 3
Q1: What was your original motivation for becoming a rural teacher? Has that changed over time?
A1: My mindset hasn't really changed. Back then, I thought teaching in the countryside wouldn't give me much sense of achievement, so I was torn a few times and seriously considered leaving. I even took the exam a few times, but every time, I just fell short by a little bit. Plus, now that I look at it, cities are actually short on middle school English teachers—so it's kind of ironic.

Q2: Have you ever thought about leaving the rural school? If so, what were the reasons, and what ultimately made you decide to stay?
A2: Yeah, a little bit. At first, being young, I thought I wanted to go to a better school closer to home—thought it'd be better for my kids' education. But after staying there for about two years, I actually started liking it. The teaching pressure is lighter there, and the students are super genuine. Plus, the teaching environment is pretty simple—there's no office politics or backstabbing. Most of us teachers are young too, and we're mostly from Xidu Town or Hengyang City. Almost all of us have cars and carpool—we take turns driving each day. When we get home, we just chat and unwind. Now I actually feel good about staying there. The only downside is that it's a bit far from home—but honestly, I end up spending more time with family now. If I were teaching in Xidu, classes would probably end at least by 6 PM. But here, if you don't do after-school tutoring, you can leave early—only if you do tutoring do you stay until 9 PM.

Q3: How do you see your future career development? Do you have plans to stay in the countryside long-term?
A3: Honestly, I'm fine with staying here in the countryside. I just want to get my professional title sorted out—that's my real goal. Right now, I don't feel like leaving at all. Being down here is comfortable, and I'm worried that if people outside think our school has a lot of open positions, they might transfer us out. But I really don't want to move right now.

Q4: Do you think the current school working environment—like hardware facilities

and teaching resources—meets teaching needs? What needs improvement?
A4: Honestly, the teaching environment still falls short. I mean, classrooms don't even have air conditioning, so in summer it gets super hot—just one fan spinning around, making kids sweat. The teaching facilities aren't great. Sure, we've got whiteboards now, but the classroom setup still needs work. There's some training, sure—but not many schools dedicate an entire semester just for lesson prep. I remember when I first started at one school, they'd never even offered English classes before. I basically had to start the whole English program from scratch there.

Q5: How's your relationship with your colleagues and school leadership? Does it affect your motivation at work?
A5: It's actually pretty good. Most of us teachers are not that far apart in age. Nowadays, young teachers make up the majority—older teachers probably only account for around 40% of the staff. It doesn't really affect motivation, but our salaries still depend heavily on academic titles. Getting a title doesn't bring much salary boost, unlike some universities where there are bonuses for high-performing classes. Here, we're not allowed to give out random rewards or extra subsidies, so there aren't many incentive programs overall. But not having rewards might slightly dampen motivation among teachers—having some kind of reward system would probably be better.

Q6: What other ways do you think the government or schools could support rural teachers to make the profession more attractive?
A6: I think it might help if they improved the reward system. Having some incentives would give teachers more motivation to teach. Also, upgrading school facilities—like installing air conditioning in every classroom—would make studying easier for students. Plus, better teaching resources would help us prepare lessons more effectively and let us learn from others' successful teaching methods.

Q7: Do you recognize the professional value of rural teachers? How does this recognition affect your willingness to stay?
A7: It's more about focusing on teaching—maybe they're just better at it from the start, so they're already ahead from day one. If these rural kids don't get motivated by young teachers down there, the gap might just keep growing. So if we want to make a change, we've gotta give our all—help those already disadvantaged kids get a good education too. This belief really shapes my decision to stay: I never forget why I became a teacher in the first place. No matter how many years you teach down here, always remember that original spark—the reason you wanted to be a teacher. Find your sense of achievement through teaching. Even if there aren't many students, every single one still matters. It's our duty to teach each kid well. Honestly, teaching down here is all about heart and conscience. No one's watching you closely—no one can really hold you accountable—but if you're a responsible teacher, you'll naturally want to do your best for every student. You just teach with integrity.

Q8: If you leave the countryside in the future, what do you think would be the most likely reason?
A8: Maybe because it's closer to home—just for convenience, family-related reasons.

Interviewee 4
Q1: What was your original motivation for becoming a rural teacher? Has it changed over time?
A1: My dad was a teacher, so I grew up with that family influence and a deep-rooted passion for education. My original motivation hasn't changed at all—I've stayed true to it the whole time.

Q2: Did you ever consider leaving the rural school? If so, what were the specific reasons? What ultimately made you decide to stay?
A2: At first, when we started working, the countryside was still underdeveloped. The key reason we stayed was that rural students tend to be quite sincere and straightforward, and these areas are also educationally underdeveloped—so these schools really need teachers like us.

Q3: Do you think the current school working environment (like hardware facilities, teaching resources) meets teaching needs?
What aspects need improvement?
A3: The teaching facilities are getting better and actually already quite good now. As for teaching resources, rural areas still need more, like psychology teachers and science/technology teachers.

Q4: Are you satisfied with your current salary and benefits? Do you think salary is a key factor affecting retention?
A4: Definitely satisfied. Given the current overall economic situation, just being able to get paid as a teacher is already pretty good. Salary definitely affects retention, but from what I've seen, the teachers who leave are mostly young ones who've passed exams to move elsewhere—not just for better pay, but also to find better career opportunities and live closer to town.

Q5: In what ways do you think the government or schools could provide more support to make teaching in rural areas more attractive?
A5: I think the government could offer more emotional support to teachers. Teachers today are under a lot of pressure, with too many non-teaching tasks—like collecting medical insurance fees. The government does support school facilities quite a bit, you can see that our school has improved a lot over the years. Getting promoted is really hard, so I hope policies will favor rural teachers more.

Q6: If you leave the countryside in the future, what do you think would be the most likely reason?
A6: Retirement. I'm already this old—I've been working in Langlong Township since

1999, so it's been almost thirty years now.

Interviewee 5
Q1: What was your original motivation for becoming a rural teacher? Has it changed over time?
A1: I tried lots of different jobs before finally settling on this one—just wanted something less stressful. Also, I'd been struggling to find something I truly wanted to do, but now I've finally found it. It took time to adjust, but my original motivation hasn't really changed.

Q2: How do you see your future career development? Do you have plans to stay in the countryside long-term?
A2: Honestly, I don't have any grand long-term plans. I just want to do my best every day I'm on this teaching job—give my all to my students. I haven't yet taught a full cohort, so I wanna experience it. I'll just finish these six years and see how it feels. No real plan to stay in the countryside long-term. Not that I don't want to—I mean, the main issue is that rural schools are facing a serious problem: too many teachers, too few students. So younger teachers like me might get reassigned or transferred eventually.

Q3: How's your relationship with your colleagues and school administrators? Does it affect your motivation at work?
A3: Pretty good. It really helps me grow. For example, when I'm preparing lessons or teaching public classes, I ask a lot of teachers for advice. And when I run into problems, I turn to them too. It has a big positive impact on my work.

Q4: Are you satisfied with your current salary and benefits? Do you think salary is a key factor affecting retention?
A4: I think it's pretty good, honestly. I'm pretty laid-back—I just need enough to cover my own expenses.

Q5: What other ways do you think the government or schools could support to make rural teaching more attractive?
A5: Honestly, right now there's hardly any attraction at all. It's not even about whether we can keep teachers anymore. Since last year, our local government has started transferring some teachers to other positions. They started testing teachers during summer break, moving some into public sector jobs because we're already completely oversaturated—there's an excess of teachers across Hengyang.

Q6: What do you think is the most likely reason you'd leave the countryside in the future?
A6: Found a better platform for growth.

Interviewee 6

Q1: What was your original motivation for becoming a rural teacher? Has it changed over time?

A1: Chosen by my parents, I didn't understand back then.

Q2: Have you ever thought about leaving your rural school? If so, what were the reasons, and what ultimately made you decide to stay?

A2: I do want to leave, but there's no way. The main reasons are the job is too far, and my home is also far away.

Q3: How do you see your future career development? Do you have plans to stay in the countryside long-term?

A3: I'm already over 40, so there's not much room for career growth—I'm fine with things as they are. I don't want to move to the city either. They wouldn't take me, and I can't even pass the exams to get in.

Q4: Do you think the current school working environment (like hardware facilities, teaching resources) meets teaching needs?

What aspects need improvement?

A4: I think the hardware facilities are a bit outdated. If it's just the school making efforts alone, it won't work—parents need to be involved too. Right now, teacher quality in rural schools is acceptable, but there's still a gap in students' starting points and parental cooperation. Working here is really tough. Parent-teacher collaboration is lacking—teachers end up doing most of the work, which puts a lot of pressure on them. Teachers often push hard on their own without much support. Our teachers' housing conditions are still stuck in the 80s—bathrooms, living spaces, everything's not suitable. This really needs serious improvement and support.

Q5: Are you satisfied with your current salary and benefits? Do you think salary is a key factor affecting retention?

A5: Honestly, given the effort I put in, I'm pretty satisfied. Of course, if it could go up even more, that'd be great—more is always better.

Q6: What do you think is the most likely reason you'd leave the countryside in the future?

A6: For people our age, I think there's basically no chance of getting promoted. If I were to leave, it'd either be due to real-life pressures or retirement. Personally, I'd rather just chill and go with the flow.

The above content consists of interview records from some teachers, which have been compiled strictly in accordance with the principle of voluntary disclosure. For the interviews with other teachers, should scholars express interest, we will systematically organize and provide the materials upon obtaining explicit consent from the respective teachers.
