# Supplementary material for: Research on influencing factors and correlation pathways of rural teachers’ retention in China
Source: Front Psychol. 2026 Jan 12;16:1728628. doi: 10.3389/fpsyg.2025.1728628 (PMC12833959; doi:10.3389/fpsyg.2025.1728628)
Supplement: Supplementary file 2 [file Data_Sheet_2.pdf]

Appendix B Expert Scoring Sheet (Factors Influencing Retention of Rural Teachers)

(i,j)	School facilities	Teaching atmosphere	Work Stress & Health	Teachers' Professional Competence	Teacher’s Occupational Quality	Institutional policy	Social status	Professional love	Wages and Benefits	Parental cooperation level	Family education culture	Student emotional engagement	Teaching resources	Student quality	Number of students	School's humanistic care
School facilities	1															
Teaching atmosphere		1														
Work Stress & Health			1													
Teachers' Professional Competence				1												
Teacher’s Occupational Quality					1											
Institutional policy						1										
Social status							1									
Professional love								1								
Wages and Benefits									1							
Parental cooperation level										1						
Family education culture											1					
Student emotional engagement												1				
Teaching resources													1			
Student quality														1		
Number of students															1	
School's humanistic care																1

Since this table has an axisymmetric structure, only the yellow region needs to be filled. The table uses a 1–5 scale (with 1 being the lowest and 5 the highest). If the row indicators(i) is more important than the column indicators(j), integer scores from 1 to 5 are assigned; if the column criterion is more important than the row criterion, reciprocal values (e.g., one-fifth, one-fourth) are used for scoring.

Score	1	2	3	4	5	1/2	1/3	1/4	1/5
Explanation of Importance	Row indicators(i) are equally important as column indicators(j).	The row criterion(i) is slightly more important than the column criterion(j).	The row criterion(i) is more important than the column criterion(j).	The row criterion(i) is very important compared to the column criterion(j).	The row criterion(i) is absolutely important compared to the column criterion(j).	The column criterion(j) is slightly more important than the row criterion(i).	The column criterion(j) is more important than the row criterion(i).	The column criterion(j) is very important compared to the row criterion(i).	The column criterion(j) is absolutely important compared to the row criterion(i).
